# Characterization of C-strain “Riems” TAV-epitope escape variants obtained through selective antibody pressure in cell culture

**DOI:** 10.1186/1297-9716-43-33

**Published:** 2012-04-20

**Authors:** Immanuel Leifer, Sandra Blome, Ulrike Blohm, Patricia König, Heike Küster, Bodo Lange, Martin Beer

**Affiliations:** 1 Institute of Diagnostic Virology, Friedrich-Loeffler-Institut, Südufer 10, 17493, Greifswald-Insel Riems, Germany; 2 Institute of Immunology, Friedrich-Loeffler-Institut, Südufer 10, 17493, Greifswald- Insel Riems, Germany; 3 Riemser Arzneimittel AG, An der Wiek 7, 17493, Greifswald-Insel Riems, Germany; 4 Institute of Virology and Immunoprophylaxis (IVI), Sensemattstrasse 293, 3147, Mittelhaeusern, Switzerland

## Abstract

Classical swine fever virus (CSFV) C-strain “Riems” escape variants generated under selective antibody pressure with monoclonal antibodies and a peptide-specific antiserum in cell culture were investigated. Candidates with up to three amino acid exchanges in the immunodominant and highly conserved linear TAV-epitope of the E2-glycoprotein, and additional mutations in the envelope proteins E^RNS^ and E1, were characterized both in vitro and in vivo.

It was further demonstrated, that intramuscular immunization of weaner pigs with variants selected after a series of passages elicited full protection against lethal CSFV challenge infection. These novel CSFV C-strain variants with exchanges in the TAV-epitope present potential marker vaccine candidates. The DIVA (differentiating infected from vaccinated animals) principle was tested for those variants using commercially available E2 antibody detection ELISA. Moreover, direct virus differentiation is possible using a real-time RT-PCR system specific for the new C-strain virus escape variants or using differential immunofluorescence staining.

## Introduction

*Classical swine fever virus* (CSFV) is one of the most important pathogens affecting domestic pigs and wild boar [1]. CSFV, together with *Border disease virus *(BDV) and *Bovine viral diarrhea virus* (BVDV), is grouped into the genus *Pestivirus * of the *Flaviviridae* family [2]. Pestiviruses are small, enveloped, single plus-stranded RNA viruses and their genome is approximately 12 300 nucleotides long and flanked by 5’-terminal and 3’-terminal non-translated regions (5’-NTR, 3’-NTR) [3]. Envelope glycoprotein E2 is the main immunogen, essential for replication [4]. Moreover, it was shown that it plays a role in viral adsorption to host cells together with other surface proteins, namely E^RNS^ and E1 [5,6]. The E2 protein forms homo- and heterodimers with the E1 protein [7-9]. So far, it is not known which regions in the E2 and E1 proteins are responsible for dimerization. The N-terminus of glycoprotein E2 displays different antigenic domains with both linear and discontinuous epitopes [10,11]. An important linear epitope located in the so-called “A domain” is the TAV-epitope consisting of the amino acids (aa) TAVSPTTLR (aa 829 to 837 in the CSFV polyprotein). This motif is highly conserved among CSFV strains but divergent in BVDV and BDV strains [12]. Several monoclonal antibodies used in CSFV diagnosis and research as well as polyclonal hyperimmune sera bind to this epitope (e.g. WH303 (Veterinary Laboratories Agency, Weybridge Surrey, UK) and A18 (IDEXX Laboratories, Shiphol-Rijk, The Netherlands)). In addition, the TAV-epitope plays a significant role in CSFV replication [13]. Especially, CSF-specific diagnostic ELISA detect antibodies directed against the conserved A-domain of the E2 structural glycoprotein, where the TAV-epitope is located [14].

Knowledge about this antibody binding site is therefore not only valuable to understand glycoprotein interactions, cell tropism, virulence, and immunology but can also be used as a target for marker vaccine and corresponding discriminatory assay development [14-16]. An example for these assays is the TAV-epitope based ELISA published by Lin et al. [17]. However, all these approaches are exclusively based on genetic engineering of marker vaccine candidates. At least in Europe, genetically modified organisms, especially the ones that enter the food chain, are viewed with caution by authorities and consumers, and this fact can lead to obstacles in both the licensing process and utilization of the final product.

In the study presented, an alternative approach was utilized that did not involve genetic engineering. In detail, C-strain “Riems” vaccine virus served as template for directed escape variant generation. This vaccine is known to be highly effective and safe after oral and intramuscular vaccination [18]. The concept was to force the vaccine strain C-strain “Riems” into TAV-epitope escape variant formation through selective antibody pressure. This pressure was triggered by monoclonal antibodies and polyclonal rabbit sera against a synthetic TAV peptide. This concept is well known for some other viruses e.g. [19,20] but so far, it has not been used for CSFV. To ensure a standardized approach and to optimize the use of possible variants, mainly commercially available monoclonal antibodies were employed. Resulting escape variants were characterized both in vitro (sequence analyses, growth characteristics, detectability with commercially available antibodies, stability, and behavior in diagnostic tests), and in vivo (safety and efficacy in challenge experiments after intramuscular administration of the variants). Moreover, concepts for genetic and serological DIVA were explored.

## Materials and methods

### Cell culture and virus propagation

Cells and viruses were grown in Dulbecco’s Modified Eagle Medium (DMEM) supplemented with 10% BVDV-free fetal bovine serum at 37°C in a humidified atmosphere containing 5% CO_2_. EFN (embryonic piglet kidney cells) and PK15 (porcine kidney) cells were obtained from the Collection of Cell Lines in Veterinary Medicine (CCLV), Friedrich-Loeffler-Institut (FLI), Insel Riems, Germany.

For cell cultivation in roller tubes, EFN cells were cultivated for one week at 37°C with DMEM containing 5% foetal calf serum (FCS) until a final cell density of 2.5 × 10^5^ cells/ mL. For virus propagation, 30 mL of a 24 h old cell suspension were incubated for one hour at 37°C with the virus isolate in the roller tube. After addition of DMEM (containing 10% horse serum) to a final volume of 300 mL, the cells were incubated for three days at 37°C on roller drums.

### Generation of polyclonal rabbit sera against CSFV E2 TAV-epitope

Two rabbits were intramuscularly vaccinated with 1 mL synthetic CSFV E2 TAV peptide (prolonged variant) at a concentration of 1 mg/mL (CTAVSPTTLRTEVVK-KLH (keyhole limpet haemocyanin) coupled) (EMC, Tübingen, Germany). To this means, 1 mg peptide was dissolved in 250 μL water and 750 μL PBS (phosphate buffered saline). One-hundred microliters of Polygen^TM^ (MVP Technologies, Omaha, NE, USA) were added as the adjuvant. The animals were boostered with 1 mL peptide solution five times at a four week interval. Serum samples were taken monthly, always one week after booster vaccination. Total serum was prepared from blood six months after the first immunization. The rabbit sera were tested in the CSFV specific E2 ELISA (HerdChek CSFV Ab, IDEXX) and in virus neutralization tests with CSFV strain Alfort/187 as described previously [9].

### Selection of E2 escape variants from cell culture supernatants

C-strain “Riems” vaccine virus batch SP 867/2 (4.5.2007) was used as the primary material. To put it under selective pressure, virus dilutions were incubated in cell culture medium for 2–3 h at room temperature with different mixtures of the E2 specific monoclonal antibodies, namely A18I (IDEXX Laboratories, Shiphol-Rijk, The Netherlands), A18B (Dr. Bommeli AG, Liebefeld, Switzerland), A18C (Cedi-Diagnostics, Lelystad, The Netherlands), HC34 (Community Reference Laboratory for CSF, Hannover, Germany) or WH303 and WH211 (Veterinary Laboratories Agency Weybridge, United Kingdom), respectively. As a standard protocol, 100 μL virus suspension was incubated with 100 μL antibody suspension containing 0.1 to 0.25 mg/mL of the respective antibody. Since the outcome of these steps was unpredictable, different concentrations and mixtures were utilized by way of trial. To impose additional selective pressure on the target region of promising candidates, polyclonal rabbit sera of CTAVSPTTLRTEVVK-peptide immunized rabbits were used in the same manner (see above). After infection of EFN cells with the virus antibody mixtures, cells were grown for three days. Supernatants were collected and cells were stained by immunofluorescence (IF) staining using the pestivirus NS3 specific C16 antibody as described elsewhere [21]. For isolation of single plaque infected wells, supernatants of positive wells were titrated on EFN cells. Subsequently, the supernatants were used for further passaging under selective E2 antibody pressure. The viruses obtained were in a first step sequenced in the corresponding regions of the E1 and E2 protein. Viruses with exchanges in the E2 TAV-epitope were used for further passaging. Promising variants with sufficient growth characteristics in cell culture were later on also sequenced in the E^RNS^ encoding region. In subsequent passages, antibody pressure was varied in terms of antibody concentration and antibody composition.

In summary, viruses were passaged several times (repeatedly) using different antibody mixtures and concentrations for selection.

Promising candidates (named in alphabetical order of their occurrence) were characterized and checked for stability after 10 to 18 passages on EFN cells in cell culture flasks or 10 passages on a roller tube system. To this means, the obtained escape variants were partially sequenced in the E^RNS^, E1, and E2 protein encoding region (method see below). Moreover, differential immunofluorescence staining using E2 TAV-epitope specific antibodies was carried out to monitor the behavior with different routine monoclonal antibodies.

### Monitoring of E2 escape variants

To monitor the behavior of the escape variants with routine monoclonal antibodies and thus test DIVA properties, cells infected with C-strain virus mutants were stained 72 hours post infection (hpi) by immunofluorescence staining and analysed using a fluorescence microscope (Olympus). Staining was carried out as described previously [21]. The following antibodies were used: A18I (IDEXX Laboratories, Shiphol-Rijk, The Netherlands), A18C (Cedi-Diagnostics, Lelystad, The Netherlands), HC34C, and C16 (provided by the Community Reference Laboratory for CSF, Hannover, Germany). Virus titres were obtained by end point titration of clarified EFN cell supernatants as described elsewhere [22]. The titres expressed as tissue culture infectious doses 50% (TCID_50_) per mL were obtained after IF staining of the cell cultures with monoclonal antibody C16 at 72 hpi.

### RT-PCR for subsequent sequencing

Relevant virus escape variants were sequenced in the E^RNS^, E1, and E2 regions using standard RT-PCR protocols [23]. Total RNA of virus-infected cell culture supernatants was extracted using the QIAamp® viral RNA mini kit (Quiagen, Hilden, Germany).

RT-PCR for sequencing was carried out with the Super Script® III One-Step RT-PCR with Platinum® Taq Kit (Invitrogen GmbH, Karlsruhe, Germany) according to the manufacturer´s instructions.

Reverse transcription was performed for 30 min at 50°C then 2 min at 94°C with primers E^RNS^-forward (5´-CAGAGACAYGAATGGAAYAAACA-3`), E^RNS^-reverse (5´-TCGGTTGACGATATTGCGTAC-3`), E1-forward      (5`-TRCCRTCRTCAGTCTGGAAT-3`), E1-reverse (5`-TGTGTAGACCACTGGCTCG-3`), E2-forward (5´-ATGGCTGTTACTAGTAACTGGG-3´) and E2-reverse (5´- TGTGTAGACCACTGGCTCG-3´). The Beacon Designer 5.0 (Premier Biosoft International, Palo Alto, USA) was used for primer selection. Oligonucleotides were provided by Eurogentec (Eurogentec GmbH, Cologne, Germany). DNA amplification was carried out with a Mastercycler ep gradient S (Eppendorf AG, Hamburg, Germany) using 42 cycles of 15 s 94°C (DNA denaturation), 30 s 57°C (annealing), and 60 s 68°C (elongation). DNA fragments were isolated from agarose gels using the QIAEX II® Gel Extraction Kit (Qiagen, Hilden, Germany).

Detection of C-strain “Riems” variants in cell culture supernatant was performed using rRT-PCR protocol as described previously [24] in an MX 3005 Pro cycler (Stratagene, La Jolla, USA) and the newly developed rRT-PCR system introduced here.

Sequencing was carried out using the BigDye® Terminator v1.1. Cyclo Sequencing Kit (Applied Biosystems, Foster City, USA). Nucleotide sequence analysis was read with a 3130 Genetic Analyzer (Applied Biosystems) and analyzed using the Genetics Computer Group software version 11.0 (Accelrys Inc., San Diego, CA, USA). All kits were used according to the manufacturer’s recommendations.

### Development of a rRT-PCR system specific for C-strain “Riems” escape variants

For differentiation of escape variants from CSFV field strains or C-strain “Riems” virus, a specific rRT-PCR system was developed. Specific primers and a FAM labelled TaqMan probe located in the TAV-epitope were designed. The forward primer Q7-TAV-for 5´ ATA-GAG-TGC-ACA-GTA-GTG-AGC-T −3` and reverse primer B5/2-TAV-rev 5´- CTC-CTG-AAG-GTC-TTT-ATG-CAC-TC −3` amplify a specific PCR product that is detected through binding of the probe C-strain-TAV-LNA-FAM 5´-AA***C*** G ***A***C ***T***C ***T*** G ***A***G ***A***A ***C*** AG-3´. The probe contains seven locked nucleic acids (LNA, presented in bold italic letters) to enable the essential melting temperature. The AgPath-ID^TM^ One-Step RT-PCR Kit (Applied Biosystems, Warrington, Cheshire, UK) was used in this protocol. The reverse transcription was performed for 10 min at 45°C followed by 10 min enzyme inactivation and polymerase activation by 95°C. The PCR was performed in 42 cycles of each 15 s 95°C (denaturation), 20 s 60°C (annealing with end-point fluorescence data collection) and 30 s 72°C (elongation).

### Challenge experiments

Three groups of five 8 to 12 week old pigs were intramuscularly immunized with escape variants Q7, S10, or O11 respectively. As control, a C-strain “Riems” vaccinated group was included and treated likewise. Pigs of the first three groups were intramuscularly immunized using 2 mL cell culture supernatant containing 1 × 10^5^ TCID_50_/mL (Q7), 1 × 10^5,25^ TCID_50_/mL (S10), and 1 × 10^5,25^ TCID_50_/mL (O11) virus, respectively. The C-strain “Riems” control group was vaccinated with one vaccine dose of 1 mL (containing 1 × 10^3.5^ TCID_50_/mL) as recommended by the manufacturer (RIEMSER Arzneimittel AG, Greifswald-Insel Riems, Germany). All pigs were handled taking into account all appropriate animal welfare regulations. The experiments were approved by the internal animal welfare officer at the FLI and the competent authority at regional level and its independent animal protection and welfare (ethic) committee. The respective reference number was LALLF M-V/TSD/7221.3-1.2-061/09.

Body temperature was measured daily and animals were monitored for clinical symptoms throughout the experiment. Serum samples were taken weekly for determination of antibody response in the IDEXX CSFV Ab ELISA (IDEXX Laboratories, Shiphol-Rijk, The Netherlands) and Ceditest CSFV E2 ELISA (Cedi-Diagnostics, Lelystad, The Netherlands). In addition, sera were tested in neutralization assays against CSFV strains “Roesrath” and “Alfort/187”. Analysis was performed as described previously [25]. For analysis of T-lymphocyte populations, whole blood samples were taken on day 21 post immunization from three animals per group. Lymphocytes were purified by Ficoll gradient centrifugation using LSM 1077 (PAA). To investigate antigen-specific T cell proliferation, lymphocytes were in vitro cultivated in the presence of UV-inactivated CSFV (C-strain, 0.1 MOI) for 5 days. Cells were stained directly ex vivo or after antigenic re-stimulation using specific mAb (AbD serotec), and FITC or PE labeled goat-anti-mouse IgG isotype secondary antibodies (dianova) in PBS containing 2% FCS, 1 mM EDTA and 0.1% NaN_3_. The following mAb were used: mouse-anti-pig CD4 (clone MIL-17), mouse-anti-pig CD8 (clone MIL-12). Labeled cells were quantitatively analyzed in a FACSCalibur flow cytometer using CELLquest software (BD Biosciences). Proliferation-specificity was calculated as stimulation index (% antigen treated / % untreated control cells).

Four weeks after vaccination, challenge infection using the highly virulent CSFV “Koslov” strain was carried out by oronasal application of 2 mL whole blood virus suspension containing 1 × 10^6,5^ TCID_50_/mL challenge virus. Three, 5, 7, and 10 days post infection, and weekly thereafter, blood and serum samples were taken. At days 3, 5, 7, and 10 post challenge infection nasal swabs were taken.

### Optimization of the DIVA concept

Besides the use of commercially available antibody ELISA that are based on E2 according to the manufacturer’s instructions, the ELISA plate was incubated for one hour at room temperature with serum of CP7_E1E2_alf_TLA [25] vaccinated pigs. The CP7_E1E2_alf_TLA vaccinated pigs do not have TAV-epitope specific antibodies since the TAV-epitope is exchanged. Afterwards the plates were washed and used following the ELISA working instructions given by the manufacturer.

## Results

### Selection of E2 escape variants from cell culture supernatants

**Figure 1:**
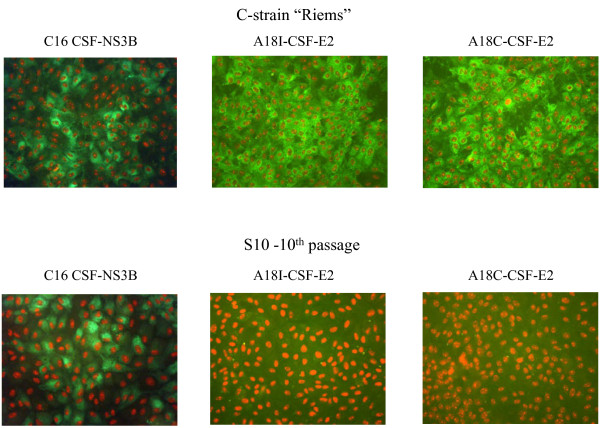
**Staining of escape variant S10 with different monoclonal antibodies.** Immunofluorescence staining of EFN cells infected with the 10^th^ passage of E2 escape variant S10 with the monoclonal antibodies C16, A18C, and A18I, the parental C-strain “Riems” virus was used as positive virus control. As conjugate the alexa Fluor®488 goat anti-mouse IgG secondary antibody was used (Invitrogen, Carlsbad, CA, USA).

After multiple passaging of C-strain “Riems”, virus variants with differences in the glycoprotein E2 encoding region were isolated from supernatants of embryonal piglet kidney cells (EFN). A first generation of E2 escape variants displayed a single nucleotide exchange in the TAV-epitope of the E2 at nucleotide position 2870, associated with a compensatory nucleotide exchange at position 2122 in the E1 encoding region. Further incubation of this variant with E2-specific neutralizing antibodies and polyclonal serum derived from TAV-peptide immunized rabbits led to the isolation of a new variant with an additional exchange in the extended TAV-epitope at nucleotide position 2889. Moreover, a third generation of E2 variants featured an additional nucleotide exchange in the TAV-epitope of the E2 protein at position 2862. In detail, the TAV-epitope of wild-type C-strainconsisted of amino acid sequence CTAVSPTTLRTEVVK. Variant B5/2 showed two exchanges resulting in sequence CTAVSSTTLRTGVVK, and variant S10 showed sequence CTVVSSTTLRTGVVK. At this point, a second compensatory exchange in the E1 protein at nucleotide position 2099 was observed. All E2 escape variants displayed an additional exchange at position 1649 in the E^RNS^ protein. All these nucleotide exchanges caused amino acid exchanges in the corresponding protein. An overview of the E2 escape variants is shown in Table 1. Immunofluorescence staining of the E2 escape variant S10 with the antibodies A18C (TAV-epitope-specific; Cedi-Diagnostics), A18I (TAV-epitope-specific; IDEXX Laboratories) and C16 (specific for nonstructural protein 3; CRL) is shown in Figure 1. Whereas E2-specific staining of the escape variant was negative even after the 10^th^ cell culture passage without antibody pressure, the parental C-strain “Riems” virus was stained positive with all E2 specific antibodies.

**Table 1:** **Stability analysis of the E2 TAV escape variants D9, B5/2, Q7, S10, and O11 using partial sequencing of viral proteins E1, E2, and E**^**RNS**^** (numbers indicate nucleotide positions differing from the parental C-strain “Riems”)**

**Escape variant**	**D9 3**^**rd**^** passage**	**B5/2 6**^**th**^**passage**	**S10-4**^**th**^**passage**	**011-5**^**th**^**passage**	**Q7-6**^**th**^**passage**	**Q7-14**^**th**^**passage**	**S10-18**^**th**^**passage**	**O11-17**^**th**^**passage**	**Resulting amino acid exchange C-strain to escape variant**
**Viral protein**									
**E**^**RNS**^	1649	1649	1649	1649	1649	1649	1649	1649	Isoleucin to Valine
**E1**	**-**	-	2099	2099	2099	2099	2099	2099	Tyrosine to Histidine
		2122	2122	2122	2122	2122	2122	2122	Aspartic acid to Glutamic acid
**E2**	-	-	2862	2862	2862	2862	2862	2862	Alanine to Valine
	2870	2870	2870	2870	2870	2870	2870	2870	Proline to Serine
	-	2889	2889	2889	2889	2889	2889	2889	Glutamic acid to Glycine

### Stability of E2 mutants

To test the stability of the acquired exchanges in the TAV-epitope, E2 escape variants were multiply passaged without antibody pressure using EFN cells. The exchange in the E2 escape variant D9 at nucleotide position 2870 was stable over more than ten cell culture passages. Escape variant B5/2 with exchanges at nucleotide position 2870 and 2889 in the E2 protein and at position 2122 in the E1 protein proved instable since the exchange at position 2889 was lost after ten cell culture passages. The escape variants Q7, S10, and O11 with nucleotide exchanges 2862, 2870, and 2889 in the TAV-epitope of the E2 protein as well as 2099 and 2122 in the E1 protein were stable over ten cell culture passages. The 14^th^ passage of S10 was week positive with the A18I antibody, single cells where stained positive (Table 2). Similarly, the 16^th^ passage of O11 was weak positive in the immunofluorescence staining with antibodies A18B and WH303. However, sequencing results of later passages of Q7 (14^th^), S10 (18^th^), and O11 (17^th^) showed no evidence for reversion of the new exchanges in the TAV-epitope and the exchanges in the E1 protein, respectively (Table 1).

**Table 2:** Stability analysis of the E2 TAV escape variants Q7, S10, and O11 using immunofluorescence staining with different E2 TAV-epitope specific antibodies or the CSFV NS3 specific antibody C16 (SC- single cells are positive, +++ strong positive)

**Escape variant**	**Q7-10**^**th**^**passage**	**Q7-14**^**th**^**passage**	**S10-10**^**th**^**passage**	**S10-14**^**th**^**passage**	**S10-16**^**th**^**passage**	**O11-10**^**th**^**passage**	**O11-14**^**th**^**passage**	**O11-16**^**th**^**passage**	**C-strain“Riems”**
**Antibody**									
A18I	-	-	-	(SC)	(SC)	-	-	-	+++
(Idexx)									
A18B	-	-	-	-	-	-	-	(SC)	+++
(Bommeli)									
WH 303	-	-	-	-	-	-	-	(SC)	+++
C16	+++	+++	+++	+++	+++	+++	+++	+++	+++

### Real-time RT-PCR system specific for C-strain-“Riems” escape variants Q7, S10, and O11

Different passages of the escape variants were tested with the specific real-time RT-PCR (rRT-PCR) system. Viruses of all passages were reliably detected with similar Cq-values (quantification cycle value) compared to the universal CSFV specific system developed by Hoffmann et al. [26] (Table 3). To test the specificity of this system, the EPIZONE reference RNA panel [24] compromising the RNA of 31 different Pestivirus strains was tested and all samples scored negative. To further evaluate the selectivity of the assay, the parental C-strain “Riems” and CSFV strain “Koslov”, both belonging to the genotype 1.1, were tested using highly positive samples with Cq-values of about 15 in the CSF specific assay. These samples gave weak positive results in the escape variant-specific rRT-PCR with Cq-values that were 20 respectively 18 Cq-values higher than in the CSF-specific rRT-PCR. Subsequent testing of 20 additional non-CSFV Pestivirus strains showed no false positive results.

**Table 3:** **Results of different C-strain escape variants and samples from the EPIZONE reference RNA panel in the TAV escape variants specific rRT-PCR system compared with the CSF specific rRT-PCR system published by Hoffmann et al., 2005**[26]

**Sample**	**TAV escape variant specific system**	**CSF specific system**
**C_strain**	No Cq.	28.3
**Eystrup91**	No Cq.	27.7
**Alfort187**	No Cq.	27.3
**Koslov1128**	No Cq.	27.2
**Brescia**	No Cq.	30.5
**Schweiz II**	No Cq.	28.4
**Pader**	No Cq.	27.2
**Bergen**	No Cq.	28
**D4886/82/Ro**	No Cq.	28.1
**Uelzen**	No Cq.	28.8
**Spante**	No Cq.	27.8
**Congenital Tremor**	No Cq.	30.4
**Q7 10**^**th**^**passage**	22.4	21.2
**Q7 14**^**th**^**passage**	21.9	20.4
**S10 10**^**th**^**passage**	21.4	20.5
**S10 14**^**th**^**passage**	20.9	19,2
**S10 17**^**th**^**passage**	21	20.1
**O11 9**^**th**^**passage**	20.9	20.1
**O11 14**^**th**^**passage**	20.2	19.5
**O11 16**^**th**^**passage**	21.6	20.1

### Animal experiment with escape variants Q7, S10, and O11

Intramuscularly immunized pigs of all groups showed full protection in challenge experiments with the highly virulent CSFV “Koslov” strain four weeks after vaccination. No CSF specific symptoms were observed. In each of the Q7, S10, or O11 vaccinated groups one animal had moderate fever for 2–4 days. The virus isolation of nasal swabs remained negative throughout the experiment in all vaccinated animals. Real-time RT-PCR results of the nasal swabs showed sporadic weak positive results in all vaccinated groups and likewise in the C-strain “Riems” vaccinated group. Real-time RT-PCR results of serum samples also showed weak positive results in the Q7, S10, and O11 vaccinated group three days post challenge infection. Animals of the unvaccinated control group showed highly positive results in the rRT-PCR of nasal swabs and serum samples from three days after challenge infection onwards and were positive in virus isolation of nasal swabs.

**Figure 2:**
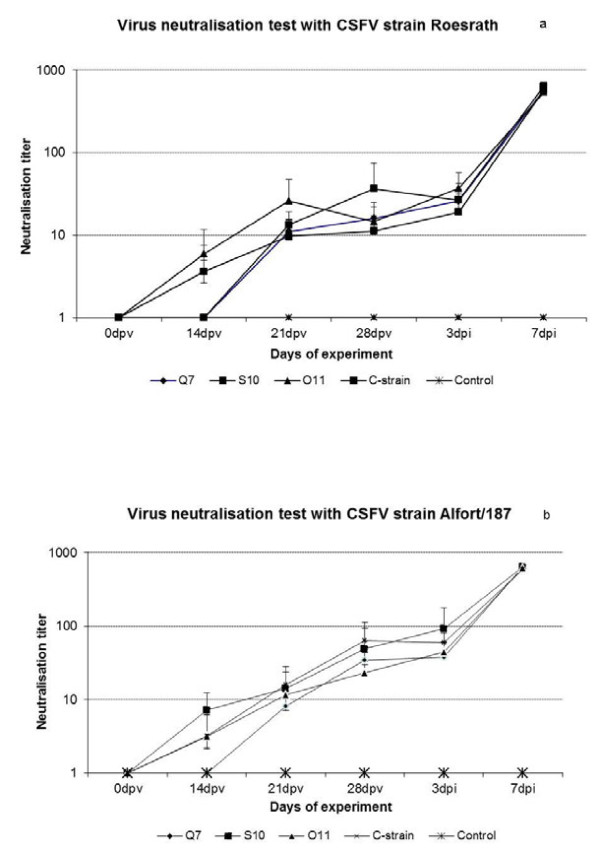
**a/b: Results of virus neutralization tests.** Virus neutralization test against CSFV strains “Roesrath” (2a) and “Alfort/187” (2b). The neutralization titers are given in a logarithmical scale.dpv: days post vaccination, dpi: days post challenge infection.

In the neutralization test (NT) against CSFV strain “Roesrath”, all groups became positive three weeks after vaccination. One animal of the S10 vaccinated group stayed negative until three days post challenge infection, but ten days post challenge infection all vaccinated animals showed neutralizing titers against CSFV strain “Roesrath” higher than 6400 neutralizing doses 50% (ND_50_) (Figure 2a). In the NT against CSFV strain “Alfort/187”, all vaccinated animals displayed neutralizing antibodies three weeks after vaccination, and ten days post infection NT titers ranging from 2400 to > 6400 ND_50_ (Figure 2b).

**Figure 3:**
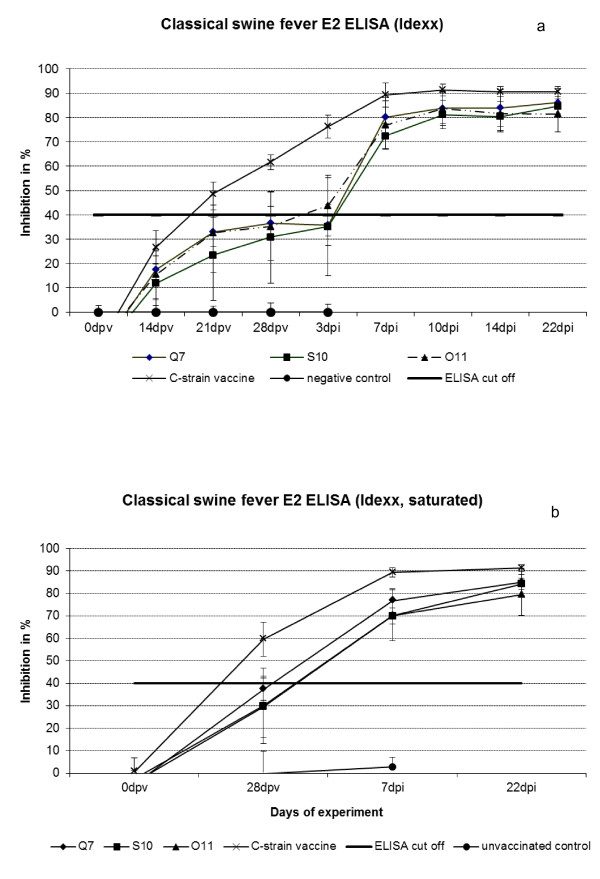
**a/b: ELISA results.3a)** E2 specific ELISA (IDEXX) of Q7, S10, O11, or C-strain “Riems” positive control immunized pigs and a mock control of unvaccinated pigs. **3b)** E2 specific ELISA (Idexx) after saturation of antigen coated plate by incubation with serum of pigs vaccinated with chimeric pestivirus CP7_E1E2alf_TLA containing E2 protein with modified TAV-epitop. dpv: days post vaccination, dpi: days post challenge infection.

In the E2 specific ELISA (IDEXX Laboratories, Shiphol-Rijk, The Netherlands) all groups vaccinated with escape variants stayed negative or showed questionable results until the day of challenge (four weeks after vaccination) whereas C-strain “Riems” vaccinated animals were clearly positive already three weeks after immunization (Figure 3a). Pre-incubation of the E2 ELISA plates with CP7_E1E2_alf_TLA [25] vaccinated pig sera also showed negative results, with about 30-37% average inhibition in all escape variant vaccinated groups. C-strain “Riems” vaccinated pigs were clearly positive four weeks after vaccination with about 60% inhibition (Figure 3b). One week after challenge infection all vaccinated pigs became positive. In the unvaccinated control group, neutralizing antibodies were not detected in the E2 ELISA nor in the NT.

To investigate alternative T-cell immunity after immunization with escape variants, peripheral blood lymphocytes were monitored three weeks after immunization and after in vitro antigen re-stimulation. Compared to untreated animals or animals vaccinated with the common C-strain, all pigs vaccinated with escape variants showed high amounts (up to 60% PBL maximum) of CD4 + CD8+ T cells in blood directly ex vivo ( Additional file 1: Figure S1a) as well as proliferation of those cells after antigenic re-stimulation with UV-inactivated CSFV in vitro. In contrast, proliferation of CD4+/CD8- T helper cells was detected after re-stimulation of lymphocytes from pigs immunized with classical C-strain “Riems” vaccine ( Additional file 1: Figure S1b).

In summary, pigs vaccinated with the different escape variants showed protection in challenge experiments comparable to C-strain “Riems” vaccinated pigs. The NT showed a similar induction of neutralizing antibodies. Clear differences could be observed in the E2 ELISA. Here, the C-strain “Riems” vaccinated pigs were positive whereas pigs vaccinated with the escape variants stayed negative or displayed questionable results. The pre-incubation of the ELISA plates with CP7_E1E2_alf_TLA vaccinated sera increased the specificity of the ELISA system slightly but did not influence the sensitivity remarkably. Pigs vaccinated with escape variants developed an alternative T cell immunity.

## Discussion

The structural protein E2 is not only the main immunogen of CSFV and therefore used in various vaccine concepts, it also plays a major role in virulence, viral adsorption to host cells and thus cell tropism, and virus replication [5,6]. Knowledge about the antibody binding sites, especially the TAVSPTTLR (aa 829 to 837) motif (TAV-epitope) in the antigenic A domain [12] can help to generate promising marker vaccine concepts and DIVA diagnostic approaches. To study the above mentioned mechanisms and to generate potential marker vaccines, genetic engineering is most often employed [14,27]. One example is the C-strain deletion mutant described by Kortekaas et al. [15]. An alternative approach involves the isolation of escape variants occurring under selective antibody pressure. While the former is much more predictable in terms of deletion sites and yields well characterized viruses by definition, the latter has the advantage of more or less naturally occurring mutations without genetic engineering. Since the escape variants develop over several passages in cell culture, it can be assumed that stability is optimized in this system, e.g. through additional compensatory or adaptive mutations. Especially the use of a well defined parental strain, i.e. a vaccine strain that was used in millions of domestic pigs and wild boars, adds safety. Moreover, since CSF vaccines will be administered to animals that enter the food chain, reservations of politicians and consumers regarding genetically modified organisms (GMO) have to be taken into account. At least in several European countries, a vaccine that is not based on a GMO would facilitate the use in case of a CSF outbreak. Furthermore, it is an advantage if one generates escape variants through the use of antibodies that are applied in commercial diagnostic test systems. This makes it more likely that animals vaccinated with these escape variants display different antibody patterns and thereby are no longer detectable with these diagnostic test systems that are fully validated and already tested for high throughput surveillance.

As a result of this study, different escape variants could be isolated from cell culture supernatants after various passages with antibody pressure. The antibody pressure was first realized in a less standardized approach with antibody mixtures by way of trial. Both, the decision on the use of a certain mixture as well as the outcome were highly incidental. In a second more targeted and interlinked approach, TAV-epitope specific polyclonal rabbit sera were used to intensify and direct the pressure. Subsequently, these escape variants were characterized both in vitro and in vivo.

Sequencing of stable variants revealed up to three exchanges in the linear TAV-epitope. These exchanges were accompanied by interesting, probably compensatory exchanges especially in the E1 and the E^RNS^ protein encoding regions. These results suggest that the TAV-epitope is probably involved in the heterodimer complex formation with the E1 protein. Similar observations were already made by Kortekaas et al. in 2010 [14], who showed that genetically modified viruses with mutations in the TAV-epitope could more efficiently replicate if compensatory mutations occurred.

In our study, adaptive exchanges were found in the E1, E^RNS^, and E2 protein, dependent on the exchanges introduced in the TAV-epitope. Interactions of glycoproteins could be one major reason for the TAV-epitope being so conserved among CSFV field strains. Of all available CSFV strains, only the GPE- vaccine strain has a slightly modified TAV-epitope with the amino acid sequence TTVSPTTLR. Like the escape variants and many CSFV field strains, GPE- possesses a glutamic acid in the E1 protein at amino acid position 583, but a tyrosine at amino acid position 575 as all other known CSFV isolates.

Most escape variants proved to be stable even after multiple passaging steps without pressure, but finally some samples showed single cell staining in immunofluorescence suggesting reversion. At least, no reversions were seen after sequencing. Whether this is due to reversion of the newly required exchanges in the TAV-epitope or is caused by unspecific binding of the E2 TAV-specific antibody, for instance because of high antibody concentration, is still unclear. One explanation could be the reversion of a minor quasispecies that would not be reliably seen in sequence analyses. Another possibility is that the isolated variant did not really start from a single plaque but from a mixture that contained the parental virus in small quantities. Given that the parental variant could have a small fitness advantage, this contamination could increase over time eventually leading to a stainable amount. Regarding stability of potential vaccine candidates, it has to be kept in mind, that industry scale vaccine production under good manufacturing practice (GMP) conditions can only use five passages of a seed virus according to the European Pharmacopoeia. Thus, stability over 10 passages is sufficient even when some properties could be lost thereafter.

An interesting question is whether repeated experiments would have led to the same escape variants. On the one hand, mutations are random effects that cannot be predicted at least in time, but on the other hand, the specific and adaptive mutations observed here seem to provide the ability to survive antibody pressure. It was observed that variants with the first exchange in the TAV epitope (nucleotide 2870) as well as with one compensatory exchange in the E1 (nucleotide 2122) occurred in independently repeated experiments. A hypothesis for this uniform behavior could be that these variants were already present in a very small quasispecies. The other mutations occurred only after several passages with different antibody compositions and concentrations. Thus, an already existing quasispecies is unlikely in this case. The use of the polyclonal anti-peptide sera was apparently necessary to obtain variants with more than two stable mutations, and the multistep approach seems to present a key factor for stable variants.

However, since repetitions and experiments in parallel were not conducted in general, these issues remain speculative.

Late generations of escape variants that showed three mutations in the TAV-epitope were tested in an animal trial in comparison with the parental C-strain “Riems” vaccine. Escape variants Q7, S10, and O11 elicited full protection against challenge infection with the highly virulent CSFV “Koslov” strain following intramuscular vaccination. Pigs vaccinated with these variants showed an enhanced T cell response that could be explained by the experimental selection pressure on E2 specific Th2 immune response. The memory T cells taken three weeks after immunization are able to expand after second antigen contact in vitro. Therefore it can be anticipated that after challenge in vivo these antigen-specific cells are able to proliferate as well and eliminate virus infected cells. In addition, it can be assumed that after challenge in vivo, antigen presentation and the following T cell activation is more effective: After re-stimulation with UV-inactivated whole virus in vitro, extracellular antigen can only be presented via cross-presentation. After challenge with living virus, antigen can be presented additionally by infected cells directly via MHC class I.

The above mentioned fever reaction was the only clinical sign and not accompanied by detectable amounts of live virus in blood, serum or nasal swabs of these pigs. Thus, protection was comparable to the one induced by the parental C-strain “Riems”. This was underlined by the very similar neutralizing antibody responses. Further animal experiments are already under execution in order to find out more about the onset of immunity, the minimum protective dosage, virus transmission, and protection after oral immunization.

Efficacy and safety of a new vaccine are of great importance, but for a possible marker vaccine, reliable and stable DIVA properties are equally pivotal. The above mentioned approach aims at using commercially available tools to differentiate infected from vaccinated pigs. The underlying assumption is that escape variants bearing modifications in the highly conserved CSFV specific linear TAV-epitope would elicit neutralizing E2-specific antibodies not recognized by the traditional E2-ELISA. First passages were not fit for purpose in this regard since animals vaccinated with these variants were positive in the commercial E2-ELISA (data not shown). Similar observations were made by Kortekaas et al. for the genetically engineered TAV mutants [15]. The concept worked in principle with later generations but still needs improvement. Pigs vaccinated with early passages of Q7, S10, or O11 stayed negative or became only questionable in the E2 specific ELISA. In the end, this would possibly work on the herd level in outbreak scenarios but is still not optimal. One approach to improve the DIVA diagnostics using the E2-ELISA was to pre-incubate the ELISA plates with sera of pigs immunized with the chimeric pestivirus CP7_E1E2_alf_TLA. These sera do not possess TAV-epitope specific antibodies but all other E2 specific antibodies, so incubation of the ELISA antigen should ensure that only TAV-epitope specific antibodies presented in the tested sera could bind. An improvement was seen, but there is still room for optimization. To further improve the DIVA principle, development of a TAV-epitope specific peptide ELISA as described by Holinka et al. in 2009 [28], Lin et al. in 2010 [17], and Kortekaas et al. in 2010 [14] would be helpful. Another possibility is the use of a double check approach where each tested sample is used in two different coated ELISA wells, one presenting the E2 antigen from the wild type CSFV and one presenting the E2 antigen from the TAV-escape variant. Thus, by analyzing both results, a clear allocation of the tested samples to wild type or TAV-escape variants should be possible.

Besides serological DIVA, direct discrimination from field viruses is possible using the rRT-PCR system introduced here. There is a certain lack of selectivity towards other genotype 1.1 strains. This problem only occurs with samples that contain high amounts of virus that would be accompanied by clinical signs if occurring with genotype 1.1 field strains. For the parental C-strain “Riems” virus, these amounts are never reached in the pig, and the vaccines would not be applied in parallel. Moreover, recent field strains detected in the European Union belong to genotype 2. Thus, problems in diagnostics arising from this minor lack of selectivity are unlikely and could, in the worst case, be solved by partial sequencing.

In summary, the escape variants obtained through selective antibody pressure could be promising marker vaccine candidates that would have the advantage that no genetic engineering was used in the design. The accompanying DIVA options have passed the proof of principle stage but still have room for optimization. Furthermore, this study might help to identify possible interacting domains of CSFV glycoproteins in the hetero dimer complex formation. The strategy to induce natural escape variants could be used in future projects to learn more about the interactions by analyzing the resulting compensatory changes.

## Competing interests

Riemser Arzneimittel AG financed the research leading to this article and is currently applying for a patent relating to the contents of the manuscript. No other competing interests exist.

## Authors' contributions

IL carried out the cell culture based techniques leading to the escape variants, investigated all samples from the related animal trials, performed sequence analyses of escape variants and their passages, developed and validated the specific real-time RT-PCR, and drafted the manuscript. SB carried out the animal trials, participated in the conception and design of the presented study, and critically revised the manuscript. UB analysed T-lymphocyte populations ex vivo and after antigenic re-stimulation. PK carried out the initial steps of escape variant formation in cell culture. HK and BL produced and characterized the escape variants on roller drums and adjusted the viruses to near industry scale production. MB conceived the study, and participated in its design and coordination and helped to critically revise the manuscript. All authors read and approved the final manuscript.

## Supplementary Material

Additional file 1 **Figure S1.** T cell response three weeks after immunization with escape variants. Compared to classical C-strain “Riems” vaccinated and unvaccinated animals, pigs immunized with escape variants show high amounts of CD4/CD8 double positive T lymphocytes directly ex vivo (A). Proliferation of double positive T cells is detectable after antigenic re-stimulation with inactivated CSFV (B). Specificity was calculated as the stimulation index (% antigen treated cells / % untreated control cells). Click here for file
