# Supplementary figures and images for: Characterization of C-strain “Riems” TAV-epitope escape variants obtained through selective antibody pressure in cell culture
Source: Vet Res. 2012 Apr 20;43(1):33. doi: 10.1186/1297-9716-43-33 (PMC3463427; doi:10.1186/1297-9716-43-33)

## Slide 1
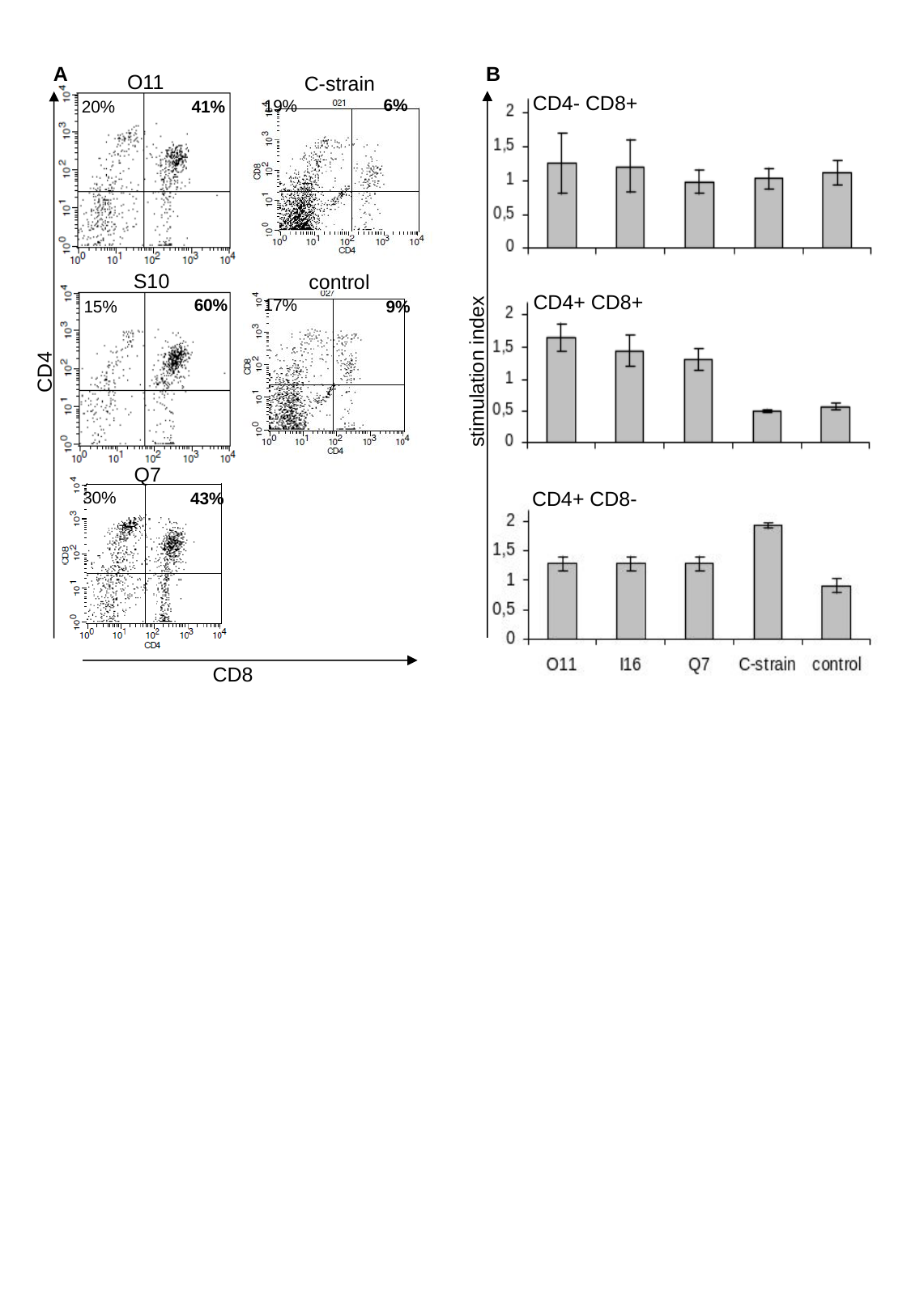

B
A
O11
C-strain
CD4- CD8+
6%
19%
20%
41%
S10
control
CD4+ CD8+
17%
60%
15%
9%
stimulation index
CD4
Q7
CD4+ CD8-
30%
43%
CD8

Supplement: Additional file 1 — Figure S1. T cell response three weeks after immunization with escape variants. Compared to classical C-strain “Riems” vaccinated and unvaccinated animals, pigs immunized with escape variants show high amounts of CD4/CD8 double positive T lymphocytes directly ex vivo (A). Proliferation of double positive T cells is detectable after antigenic re-stimulation with inactivated CSFV (B). Specificity was calculated as the stimulation index (% antigen treated cells / % untreated control cells). [file 1297-9716-43-33-S1.ppt]
